# The Numbers of Practitioners and Students

**Published:** 1916-02-05

**Authors:** 


### THE NUMBERS OF PRACTITIONERS AND STUDENTS.

A special interest is attached to the figures which show the numbers of new medical practitioners and new medical students registered in 1915. Existing conditions relative to the medical profession are so unusual that there has been legitimate anxiety lest the claims of the moment should prejudice the needs of the future, and very gloomy prophecies have been cultivated of a population smitten by epidemics and destitute of efficient assistance. The published figures do little to support these expectations. As a matter of fact, the number of practitioners registered in 1915 is considerably above the average. Probably the chances of a commission in the R.A.M.C. have stimulated the fifth-year students to push forward to qualification in larger numbers than would otherwise have been the case, and there is every reason to expect that fourth-year students will, during the current year, respond in similar fashion. Hence the immediate future would seem to be secure even when allowance is made for the losses necessarily inflicted by the war, and the authorities may plead the figures in justification of their decision to advise senior students to complete the curriculum.

Still more anxiety was felt in regard to the supply of students for the future, and the official proposal to permit the junior students to enter the combatant ranks of the Army produced many anticipations of disaster. Yet here again the figures appear to contradict the prophets, for the registrations exceed the average of the previous five years by more than 450 entries. Some part of this increase is doubtless due to additions to the number of women students, but even the most liberal allowance on this point will not account for anything like the total increase. On the other hand, it must be recognised that not all those whose names appear on the register are at the moment actually engaged in medical study. Many of them—the exact figures are not yet known—are either serving with the Army or are in training for this duty. Yet, taking the facts as they stand, there is little reason for the pessimistic views which were so loudly proclaimed. In these columns we have always anticipated that the prospects offered by the profession could be relied on to secure a reasonable number of disciples, and we have been unable to sympathise with the censures which were so freely applied to the War Office. The actual figures render rather ridiculous the statement of the seer who announced that the policy of the authorities would "leave disease an unchecked career throughout the land." Curiously enough, a similar line of criticism is now being advanced by some representatives of the dental profession, one of whom has recently told the public that with dental students taken by the Army "the succeeding generation will be decimated by tuberculosis . . . because their teeth have been allowed to rot in their jaws." The educational value of a scientific training is just now a popular theme, but it hardly finds justification in the public proclamations of some of the experts. That everything during the next few years will be smooth sailing in the supply and distribution of medical practitioners is hardly possible, but some estimates of the difficulty have been excessive. And with organisation and good will we believe the position will prove to be far from desperate.

